# Identification and Functional Characterization of a Novel SEMA3A Exon Deletion Variant in Kallmann Syndrome

**DOI:** 10.1002/mgg3.70190

**Published:** 2026-01-14

**Authors:** Shaolian Zang, Shasha Zhou, Qingxu Liu, Xiaoqin Yin, Pin Li

**Affiliations:** ^1^ Research Center for Clinical Diagnosis and Treatment of Sexual Development Disorders, Shanghai Children's Hospital, School of Medicine Shanghai Jiao Tong University Shanghai China; ^2^ Department of Endocrinology, Shanghai Children's Hospital, School of Medicine Shanghai Jiao Tong University Shanghai China

**Keywords:** cell migration, GnRH, Kallmann syndrome, pathogenesis, SEMA3A

## Abstract

**Background:**

Kallmann syndrome (KS) is a genetic disorder characterized by impaired reproductive system and olfactory development. This study aimed to identify a novel variant of *SEMA3A* in a KS patient and explore its potential pathogenic mechanism.

**Methods:**

A gene panel was used to identify potential pathogenic mutations. Wild‐type and mutant *SEMA3A* overexpression plasmids were constructed. Western blotting, RNA sequencing, and cell migration were performed to assess the effects of *SEMA3A* gene variations on GnRH neuronal migration.

**Results:**

A novel heterozygous mutation in the *SEMA3A* gene (NM_006080.3: exon 6–9 deletion) was identified in the proband, as well as in his father and sister. The spatial structure of the *SEMA3A* mutant protein was relatively looser. In vitro experiments revealed that *SEMA3A* mutation reduced SEMA3A expression and inhibited GnRH neuronal migration. RNAseq analysis revealed that the expression of 76 genes was upregulated and that of 104 genes was downregulated after *SEMA3A* mutation. The altered gene clusters were enriched mainly in cell migration, male gonad development, motor proteins, and neuron synapses.

**Conclusions:**

In this study, we identified a novel variant of *SEMA3A* in a KS patient and verified its function. These findings expand the mutation spectrum of the *SEMA3A* gene and offer a theoretical basis for the clinical diagnosis of KS.

## Graphical abstract

The study identified a novel *SEMA3A* exons 6–9 deletion variant in Kallmann syndrome that impairs GnRH neuronal migration and alters cell migration, gonad development, and synaptic pathways. The study expands mutation spectrum and offers mechanistic insights for clinical diagnosis of Kallmann syndrome.

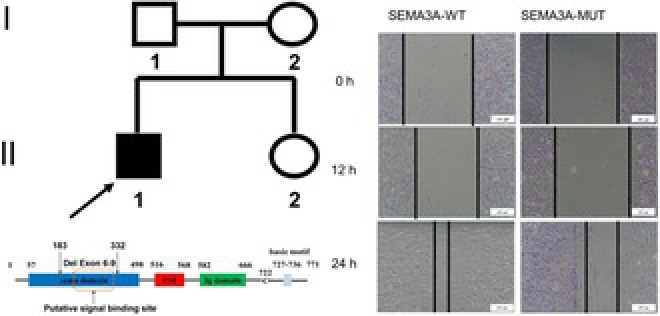

## 1 Introduction

Adolescence is a critical phase in human growth and development. Both the onset of puberty and reproductive capacity depend on the precise establishment of axonal projections by hypothalamic gonadotropin‐releasing hormone (GnRH) neurons to the median eminence (ME) and the subsequent secretion of GnRH. During embryonic development, GnRH neurons originate in the nasal placode, migrate along the terminal nerve, and are guided by olfactory ensheathing cells to the medial preoptic area of the hypothalamus. Upon reaching this destination, GnRH neurons stop migrating and extend axonal projections to form synaptic connections with the ME. At this site, complex neuron–glia–endothelial (NGE) interactions regulate GnRH homeostasis, thereby activating the hypothalamic–pituitary–gonadal (HPG) axis (Giacobini et al. 2014; Hanchate et al. 2012). Disruptions in GnRH neuronal migration may result in insufficient GnRH secretion, leading to delayed puberty or its complete absence.

Idiopathic hypogonadotropic hypogonadism (IHH) is a developmental disorder that affects sexual maturation and is characterized by delayed puberty or gonadal dysfunction caused by impaired synthesis, secretion, or action of gonadotropin‐releasing hormone (GnRH) (Bianco and Kaiser 2009). When IHH coexists with anosmia or hyposmia, it is termed Kallmann syndrome (KS), accounting for approximately 60% of cases (Zhou et al. 2022). The genetic mechanisms underlying IHH are multifactorial and involve mutations in genes critical for GnRH neuronal differentiation and migration, olfactory axon guidance, neuroendocrine homeostasis, and gonadotropin synthesis/release (Young et al. 2019). Patients with KS commonly exhibit a wide range of clinical phenotypic features. These include reproductive system abnormalities, such as cryptorchidism and micropenis, as well as extragonadal manifestations, such as cleft lip/palate, skeletal dysplasia, sensorimotor ataxia, and renal agenesis (Bonomi et al. 2018). Genetic molecular diagnosis, conducted through prenatal diagnosis and preimplantation genetic testing, is the most effective strategy for reducing the risk of transmitting KS to the next generation. Early diagnosis, combined with appropriate dietary supplementation and medical interventions, can help prevent complications associated with KS. For instance, various hormone therapy protocols and timelines have been proposed for patients with Duchenne muscular dystrophy (DMD) (Sodero et al. 2025). Research has indicated that the primary treatment modalities for patients with KS include surgical intervention, low‐dose testosterone therapy (such as dihydrotestosterone or testosterone esters), gonadotropin therapy (luteinizing hormone/follicle‐stimulating hormone), human chorionic gonadotropin (hCG), and low‐dose estrogen or progesterone therapy. The selection of a specific treatment plan and the corresponding medication dosage are determined on the basis of the patient's sex and age (Castro et al. 2025; Rhys‐Evans et al. 2024; Yadav et al. 2025). The primary objectives of therapeutic intervention for KS are the promotion of the development of secondary sexual characteristics, the preservation of bone density and muscle mass, and the potential restoration of fertility.

To date, nearly 50 genes have been associated with IHH, including approximately 20 genes specifically associated with KS. Pathogenic variants in genes such as *CHD7*, *FGF8*, *FGF17*, *FGFR1*, *HS6ST1*, *NSMF (NELF)*, *PROK2*, *PROKR2*, and *WDR11* are known to cause both KS and normosmic IHH (nIHH). Additionally, pathogenic variants in *ANOS1 (KAL1)*, *CCDC141*, *FEZF1*, *IL17RD*, *SEMA3A*, *SEMA3E*, and *SOX10* are primarily implicated in KS. Furthermore, pathogenic variants in *GNRH1*, *GNRHR*, *KISS1*, *KISS1R (GPR54)*, *TAC3*, *and TACR3* are predominantly associated with nIHH (Kaluzna et al. 2024; Topaloglu 2017). In our previous retrospective study on IHH, missense variants represented the primary genetic cause of the condition, with *FGFR1*, *PROKR2/PROK2*, and *KAL1* identified as the main pathogenic genes (Liu et al. 2022).

However, these genetic variations account for approximately 50% of the cases, suggesting that other factors may also play significant roles in the remaining cases (Boehm et al. 2015; Cangiano et al. 2021). Previous studies have shown that SEMA3A‐deficient mice exhibit abnormalities in the migration of olfactory/vomeronasal axons and GnRH neurons (Cariboni et al. 2011). *SEMA3A* (OMIM: 603961) belongs to the class 3 semaphorin family, a group of signaling proteins that encode secreted factors (Yazdani and Terman 2006). *SEMA3A* mutations are responsible for approximately 6% of KS cases. However, the precise molecular mechanisms underlying this association remain unclear, highlighting the need for further investigation.

## 2 Methods and Materials

The patient in this study was a 15‐year‐old male who was referred to the Endocrinology Clinic of Shanghai Children's Hospital because a small penis was observed at birth. Following a comprehensive examination, he was diagnosed with KS. Following this diagnosis, his parents and sister were invited to the hospital for further familial assessment. All participants signed informed consent forms, and the study received approval from the Ethics Committee of Shanghai Children's Hospital (Approval No. 2021R092‐E03). Peripheral blood samples from all family members of the patient were collected in anticoagulant tubes and centrifuged at 3000 *g* for 10 min, after which the pelleted blood cells in the lower layer were transferred to 2 mL EP tubes.

### 2.1 Gene Panel

DNA was extracted from the blood samples using a commercial kit (QIAGEN, Germany). After quality assessment, the genomic DNA was enzymatically digested into fragments. Subsequent procedures included end repair, A‐tailing, adapter ligation, and PCR amplification, which were performed in sequence to construct a DNA library. Following an additional quality check, target gene and splicing region sequences were captured and enriched using custom‐designed probes for high‐throughput sequencing. The coverage of the target region reached ≥ 99%, with an average sequencing depth of ≥ 200×. After stringent quality control measures were applied to the raw sequencing data, low‐quality reads were filtered out. The remaining high‐quality short reads were aligned to the human genome reference sequence, enabling the detection and characterization of variant sites. The annotation of the gene set was performed on human GRCh37/hg19.

### 2.2 Cell Culture

GN11 cells, an immature GnRH neuronal cell line (Hoffmann et al. 2016), were utilized as a cell model for functional studies. The cells were maintained in DMEM supplemented with 10% fetal bovine serum, 100 U/mL penicillin, and 100 μg/mL streptomycin. The cultures were incubated at 37°C under a humidified atmosphere of 5% CO_2_.

### 2.3 Plasmid Construction and Cell Transfection

Wild‐type and mutant eukaryotic expression vectors for the *SEMA3A* gene were constructed and packaged by YiXueSheng Biosciences Inc. (Shanghai, China). The plasmid was extracted and amplified using a plasmid extraction kit (TIANGEN, China). Cells were seeded into a 6‐well plate at a density of 5 × 10^5^ cells per well and transfected with the plasmid after 12 h of culture. Plasmid DNA was diluted in an appropriate buffer and mixed with Lipofectamine 3000 reagent (Thermo Fisher, USA) according to the manufacturer's instructions. The mixture was incubated at room temperature for 15 min before being added to the 6‐well plate and gently mixed. The transfected cells were subsequently cultured in a humidified incubator at 37°C with 5% CO_2_ for further experiments.

### 2.4 Wound‐Healing Assay

Approximately 2.5 × 10^5^ GN11 cells were transfected into each well to ensure that the cells reached confluence by the following day. A 200 μL pipette tip was used to create three perpendicular lines on the cell monolayer in each well. The cells were gently washed three times with 1× PBS to remove any detached cells, after which serum‐free medium was added. The cells were cultured at 37°C in a 5% CO_2_ incubator. The samples were collected at 0, 12, and 24 h, and five fields of view were randomly selected for imaging. The images were analyzed using ImageJ software.

### 2.5 Transwell Assay

In this experiment, 8 μm Corning Transwell chambers (24‐well plates) were used. The basement membrane was hydrated with 1 × PBS. The cells were serum‐starved for 12 h in serum‐free medium prior to preparing the cell suspension. GN11 cells transfected with the plasmids were digested using 0.25% trypsin, after which complete culture medium was added to stop digestion. The mixture was subsequently centrifuged, the supernatant was discarded, the cells were washed twice with 1 × PBS, and the cells were resuspended in serum‐free medium at a density of 5 × 10^5^ cells/mL. Subsequently, 600 μL of DMEM containing 10% FBS was added to the lower chamber of the Transwell insert, while 100 μL of the cell suspension was added to the upper chamber. After incubation for 24 h, the Transwell chamber was removed, the upper culture medium was discarded, and the chamber was washed twice with 1 × PBS. The cells were then fixed with 4% paraformaldehyde for 30 min, air‐dried, and stained with 0.1% crystal violet for 20 min. Finally, the chamber was removed, and five random fields of view were imaged for subsequent analysis using ImageJ software.

### 2.6 Real‐Time Quantitative PCR (qPCR)

Genomic DNA was extracted from peripheral blood, and the relative copy number of *SEMA3A* was quantified using qPCR. Amplification was performed on a real‐time thermal cycler (Bio‐Rad, USA). The reaction mixture consisted of 1 μL forward primer, 1 μL reverse primer, 5 μL SYBR Green PCR Master Mix (AG, China), and 3 μL genomic DNA template in a final volume of 10 μL. The PCR program was as follows: initial denaturation at 95°C for 30 s, followed by 40 cycles of denaturation at 95°C for 5 s and annealing/extension at 60°C for 30 s, with a final hold at 4°C. Exon 10 was used as the reference genomic region. The primers (Tables S1 and S2) were synthesized by Sangon Biotech Co. Ltd. (Shanghai, China).

### 2.7 Western Blotting

The SEMA3A wild‐type and mutant plasmids were transfected into GN11 cells using Lipofectamine 3000 (Invitrogen, USA). After 72 h of incubation, the cells were harvested and transferred to 1.5 mL EP tubes. Afterward, 80 μL of protein lysis buffer supplemented with protease inhibitors (protease inhibitor: protein lysis buffer = 1:100) (Thermo Fisher Scientific, USA) was added. The mixture was vortexed gently and incubated on ice for 30 min. Protein separation was achieved by sodium dodecyl sulfate–polyacrylamide gel electrophoresis (SDS–PAGE) using a 10% gel, followed by transfer onto a polyvinylidene fluoride (PVDF) membrane (Millipore, USA). Immunoblotting was performed using a rabbit monoclonal anti‐SEMA3A antibody (1:1000; #A22204; ABclonal, China) and a rabbit monoclonal anti‐DYKDDDDK tag antibody (1:10,000; #PTM‐5577; PTM Biolabs Inc., China). All experiments were repeated at least three times to ensure reproducibility.

### 2.8 RNA Sequencing

The *SEMA3A* wild‐type and mutant plasmids were transfected into GN11 cells using Lipofectamine 3000 (Invitrogen, Carlsbad, CA, USA). After 72 h of transfection, the cells were harvested and collected in 1.5 mL EP tubes. Total RNA was extracted from the collected cells using TRIzol reagent according to the manufacturer's protocol. The quality and concentration of RNA were assessed using a NanoDrop 2000 spectrophotometer (Thermo Scientific, USA), while RNA integrity was evaluated using an Agilent 2100 Bioanalyzer (Agilent Technologies, USA). The transcriptome library was subsequently constructed using the VAHTS Universal V5 RNA‐seq Library Prep Kit (Vazyme Biotech Co. Ltd.) following the manufacturer's instructions. Sequencing was performed on the Illumina NovaSeq 6000 platform, generating 150 bp paired‐end reads. Each sample yielded approximately 50 million raw reads, which were processed using fastp software to obtain high‐quality clean reads. Reference genome alignment was conducted using HISAT2 software, and gene expression levels were quantified as fragments per kilobase of transcript per million mapped reads (FPKM). Differential expression analysis was performed using DESeq2 software, identifying differentially expressed genes (DEGs) with a *p* value < 0.05 and a |log2FC| ≥ 1. Enrichment analyses for Gene Ontology (GO) and Kyoto Encyclopedia of Genes and Genomes (KEGG) pathways were performed on the identified DEGs. Transcriptome sequencing services were provided by Shanghai OE Biotech Co. Ltd. (Shanghai, China).

### 2.9 Statistical Analysis

The results were subjected to statistical analysis using SPSS version 13.0, with intersample differences evaluated via an unpaired t test. Graphical representations were generated using GraphPad Prism version 8.0.1. *p* < 0.05 was considered to indicate statistical significance.

## 3 Results

### 3.1 Identification of a Novel SEMA3A Variant in Patients With KS

**FIGURE 1:**
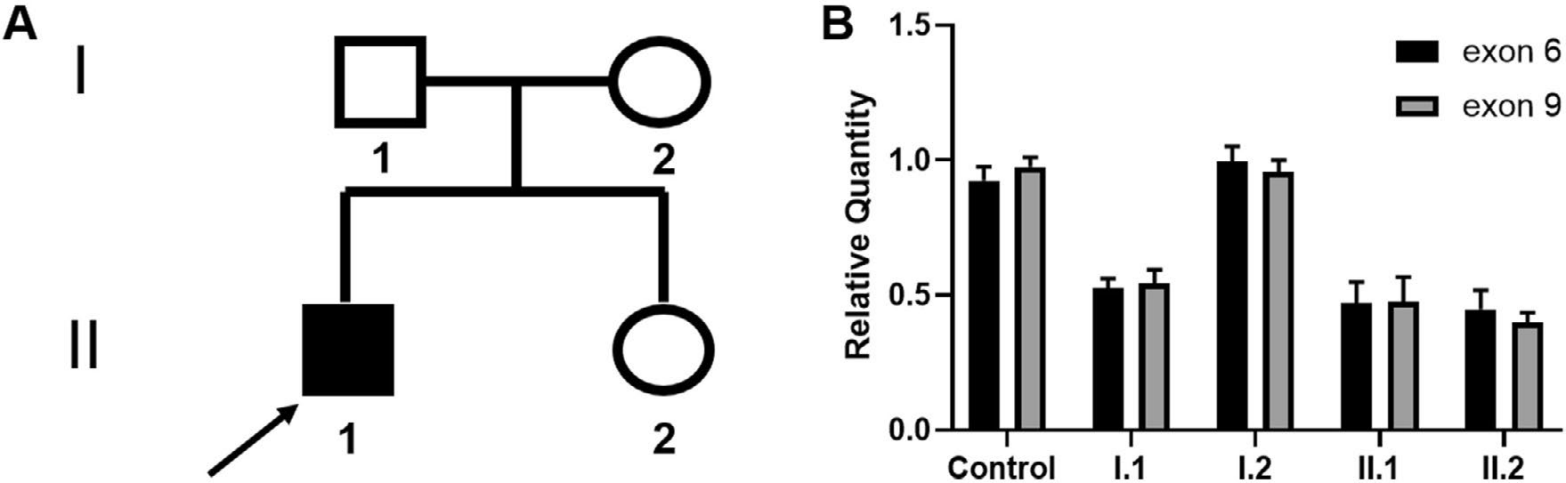
SEMA3A exon deletion in a family with KS. (A) Pedigree of the family with heterozygous SEMA3A deletion. The proband is Subject II‐1 (arrow). The affected father (I‐1) and sister (II‐2) are also heterozygous for the same deletion. The solid symbols indicate clinically affected subjects. The circles represent female family members, and the squares represent male family members. (B) Quantitative PCR validation of the SEMA3A heterozygous deletion. The black column indicates exon 6, and the gray column indicates exon 9, which was deleted in Subjects I‐1, II‐1 and II‐2 (see A). The results are presented as the means ± SDs of three independent experiments.

We identified a heterozygous variant of *SEMA3A* characterized by the deletion of exons 6–9 in a male proband diagnosed with KS (Figure 1A). The clinical characteristics of the proband are summarized in Table 1. Notably, the proband (II:1), who carries the deletion variant of exons 6–9, has been diagnosed with KS. However, his father (I:1) and sister (II:2), who also have this deletion variant, exhibited normal puberty and fertility. Furthermore, his mother (I:2) does not carry the variant and is unaffected. Heterozygous deletions were confirmed by quantitative real‐time PCR, with exon 10 serving as the control. Real‐time PCR analysis revealed that the heterozygous *SEMA3A* deletion was present in two relatives of the proband affected by KS, suggesting that this *SEMA3A* deletion cosegregates with the KS phenotype (Figure 1B).

**TABLE 1:** The results of auxiliary examination on the KS proband.

Clinical examination	Results	Reference range
Height/weight (cm/kg)	160/61.4	/
Reproductive phenotype at diagnosis	Hypogonadism Testicle: 4–5 mL Penis: 3 × 1.5 cm	/
Smell	Anosmia	/
Nasopharyngeal MRI	Bilateral olfactory bulb and olfactory tract were not clear	/
Testicular b‐ultrasonography	Microtesticle	/
Breast b‐ultrasound	Male breast development	/
Hormone assay		
T (nmol/L) Basal → Stimulus	1.05 → 9.96	6.0–27.0
DHT (pg/mL) Basal → Stimulus	42.74 → 222.44	70.30–1260.90
LH (IU/L) Basal → Stimulus	1.11 → 16.9	0.80–7.60
FSH (IU/L) Basal → Stimulus	2.43 → 9.36	0.70–11.10

### 3.2 Bioinformatic Analysis

**FIGURE 2:**
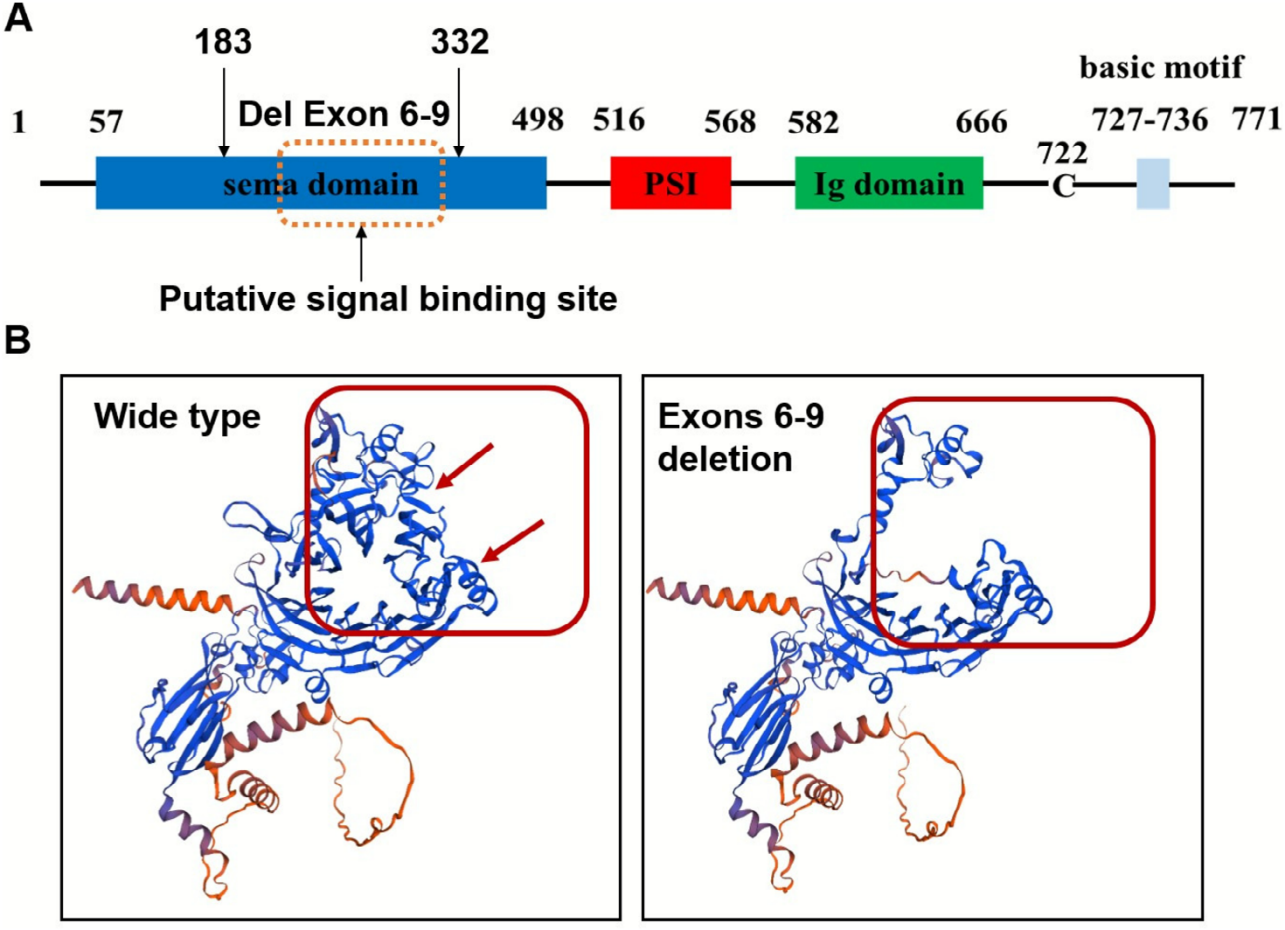
SEMA3A mutant protein domain and protein structure prediction. (A) The domain of the SEMA3A protein. Blue, sema domain; red, PSI; green, Ig domain; gray, basic motif. (B) Prediction of the protein structure of wild type and mutant SEMA3A.

Bioinformatics analysis was used to predict the pathogenicity of the de novo variant of *SEMA3A*. As illustrated in Figure 2A, this deletion variant is located within the SEMA domain and is situated at both putative signal binding sites. Research has indicated that this region plays a crucial role in SEMA3A signaling (Siebold and Jones 2013). To assess the impact of this mutation on the SEMA3A protein, we utilized SWISS‐MODEL to construct a three‐dimensional model of the SEMA3A homodimer and visualized the mutation‐related region using DeepView. The results, as depicted in Figure 2B, revealed that the overall spatial structure of the mutant protein was relatively looser than that of the wild type. Therefore, on the basis of the bioinformatics analysis, we hypothesize that the novel *SEMA3A* mutation is likely to have pathogenic significance.

### 3.3 Functional Analysis

**FIGURE 3:**
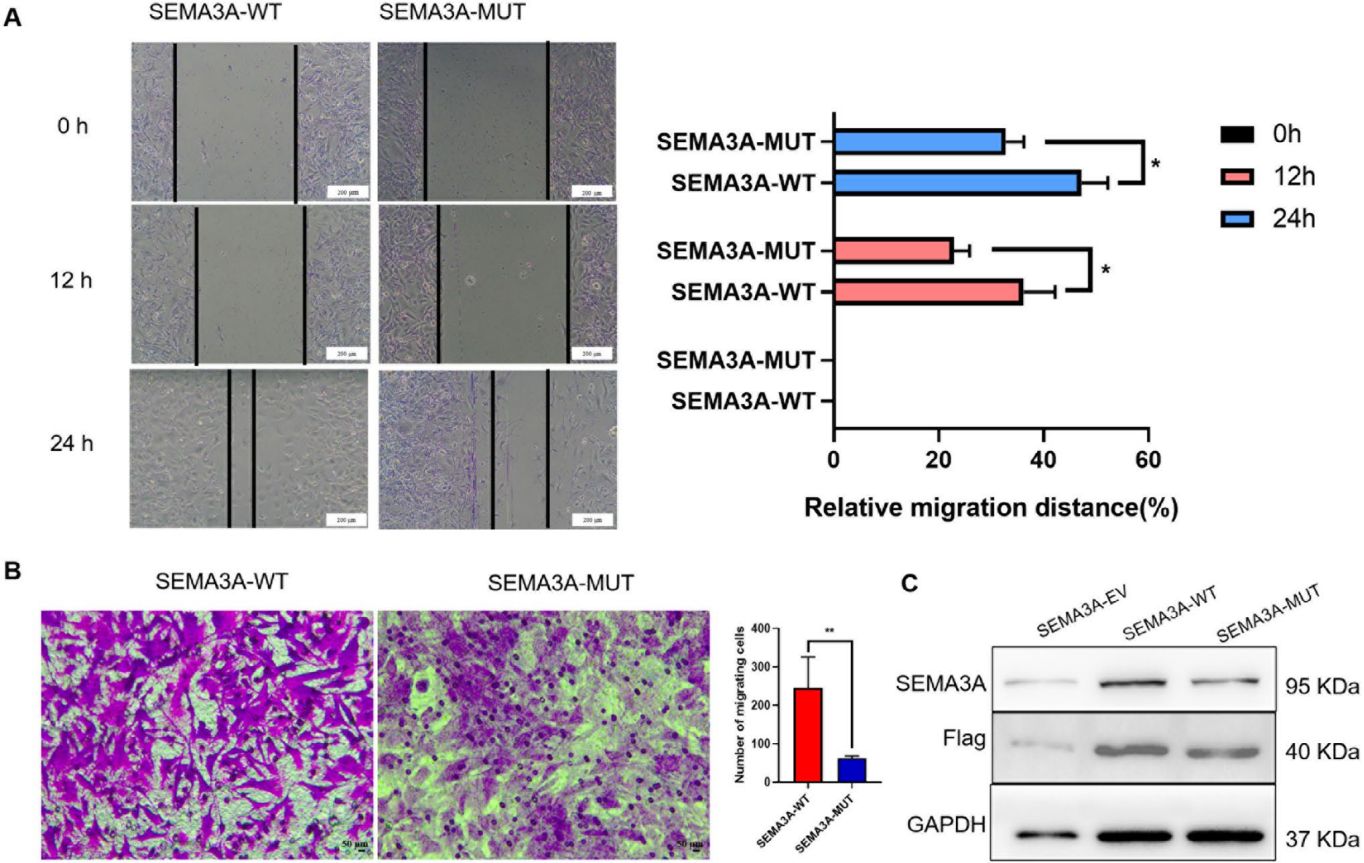
KS‐associated SEMA3A mutations disrupt the ability of SEMA3A to stimulate GN11 cell migration. (A) Left: Representative images of wound‐healing assays performed on GN11 cells expressing WT or mutant SEMA3A proteins. The continuous lines indicate the borders of the wound. Right: Statistical analysis of the results of the transwell assay (*n* = 3). The images were taken at the same magnification (magnification, 100×. Scale bars, 200 μm). (B) Left: Representative images of the Transwell assay performed on GN11 cells expressing WT or mutant SEMA3A proteins. Right: Statistical analysis of the results of the transwell assay (*n* = 7). The images were taken at the same magnification (magnification, 200×. Scale bars, 50 μm). (C) In the SEMA3A‐WT and SEMA3A‐MUT groups, a schematic of the SEMA3A‐Flag protein WB in GN11 cells (detected using SEMA3A and Flag antibodies, respectively). **p < 0.01, *p < 0.05. Unpaired two‐tailed Student's *t*‐test was used.

We generated Flag‐tagged SEMA3A constructs, including exon 6–9 deletion mutants and wild‐type plasmids. These plasmids were individually transfected into GN11 cells. In the scratch test, the relative migration distance of GN11 cells in the SEMA3A‐MUT group was significantly reduced at both 12 and 24 h. In contrast, the SEMA3A‐WT group exhibited a significantly greater relative migration distance at both time points (*p* < 0.05) (Figure 3A). These observations were consistent with those of the transwell assay, which revealed that after 24 h, the number of migrating cells in the SEMA3A‐MUT group significantly decreased (*p* < 0.01) (Figure 3B). Given the critical role of SEMA3A in neuronal cell migration, we further investigated the protein expression levels of SEMA3A in GN11 cells. As shown in Figure 3C, the protein expression level of SEMA3A was significantly decreased in the SEMA3A‐MUT group. In this study, GN11 cells were transfected with a eukaryotic expression plasmid encoding FLAG‐tagged SEMA3A, including both the wild‐type and mutant forms. The expression of SEMA3A markedly increased, as confirmed by Western blot analysis using a FLAG‐specific antibody. The mutant form of the SEMA3A protein was found to influence the migration of GN11 cells.

### 3.4 Transcriptome Sequencing Analysis of *SEMA3A* Exon Deletions

**FIGURE 4:**
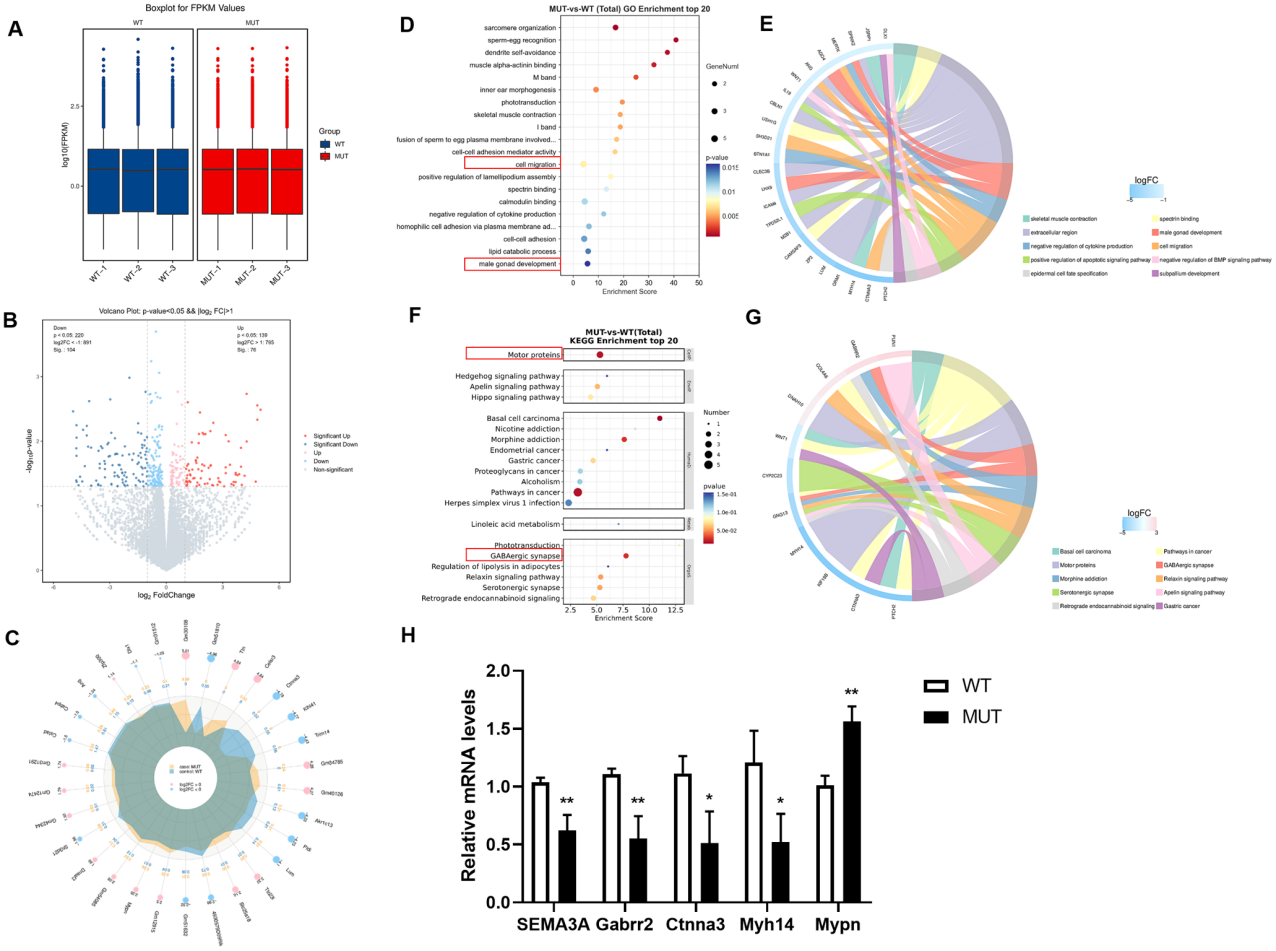
Transcriptome sequencing analysis after SEMA3A exon deletion. (A) Box plots of the FPKM values of each sample gene. (B) Volcano plot illustrating differential gene expression across groups. Genes with nonsignificant differences are indicated in gray, while those with significant differences are highlighted in red and blue. The horizontal axis represents the log2(fold change), and the vertical axis denotes the −log_10_ *p* value. (C) Radar map of the expression levels of 30 differentially expressed genes with minimal *p* values. (D) GO enrichment analysis of the top 20 genes shown in the bubble map. (E) Chord chart of GO enrichment in the top 10 genes. (F) KEGG enrichment analysis of the top 20 genes shown in the bubble map. (G) Chord chart of the top 10 enriched KEGG pathways. (H) qRT–PCR was used to validate the mRNA levels of SEMA3A, Ctnna3, Gabrr2, Myh14 and Mypn. The results are presented as the means ± SDs of three independent experiments. **p < 0.01, *p < 0.05. Unpaired two‐tailed Student's *t*‐test was used.

GN11 cells transfected with *SEMA3A* mutant plasmids (specifically, exons 6–9 deletion) and wild‐type plasmids were subjected to reference‐based transcriptome sequencing on the Illumina NovaSeq 6000 platform. Following quality control, high‐quality data were obtained, with an average Q30 > 94% and more than 49 million reads per sample. The dispersion of the distribution of differentially expressed genes (DEGs) was consistent across samples (Figure 4A). Differential expression analysis using DESeq2 (|log2FC| ≥ 1, *p* < 0.05) revealed 180 significantly DEGs, comprising 76 upregulated genes and 104 downregulated genes (Figure 4B). The upregulated genes included Mypn and Fam227b, whereas the downregulated genes included Sh3d21, Dlx1, Cabp4, Lhx9, and Ccdc106 (Figure 4C). Notably, Gene Ontology (GO) enrichment analysis revealed that the DEGs associated with cell migration and male gonad development were significantly enriched (Figure 4D). Specifically, genes involved in the cell migration pathway included Ang, Ctnna3, Mertk, and Sh3d21, whereas those related to male gonad development included Ago4, Lhx9, and Spink2 (Figure 4E). Furthermore, Kyoto Encyclopedia of Genes and Genomes (KEGG) enrichment analysis demonstrated significant enrichment of DEGs in the motor proteins and GABAergic synapse signaling pathways (Figure 4E). Notably, the key genes involved in the motor protein signaling pathway were Dnah10, Kif19b, and Myh14, whereas the genes contributing to GABAergic synapse signaling were Gabrr2 and Gng13 (Figure 4F). qRT–PCR was used to further investigate the relationships between *SEMA3A* gene mutation and motor proteins and GABAergic synapses. SEMA3A, Ctnna3, Gabrr2 and Myh14 mRNA expression decreased significantly. In addition, Mypn mRNA levels were increased (Figure 4G). Collectively, the results of this study provide multiomics evidence for the dysregulation of signaling pathways induced by mutations in the functional domain of SEMA3A.

## 4 Discussion

In this study, a gene panel and functional experiments were used to identify a novel gene variant of *SEMA3A* associated with KS. These findings expand the spectrum of mutation‐related genes in KS. Furthermore, this study elucidated the relationship between *SEMA3A* and the phenotypic manifestations observed in these patients. The alteration of the *SEMA3A* gene is most frequently associated with anosmia, delayed puberty, micropenis, and low serum testosterone in males with KS. Moreover, we explored the cellular mechanisms through which SEMA3A regulates GnRH neuron migration. This variant has not been cataloged in public databases such as ClinVar. Tools such as SpliceAI and CADD are designed to evaluate variations near splice sites, such as single‐nucleotide variants or small insertions and deletions, but they are not suitable for analyzing the deletion of entire exons. Given that this variant results in the deletion of multiple exons, pathogenicity assessment cannot be performed using the aforementioned computational tools. Instead, the evaluation of its pathogenic potential should be based on a comprehensive analysis incorporating clinical phenotypic data and functional studies.

In this study, we identified a heterozygous mutation involving the deletion of exons 6–9 in the *SEMA3A* gene. The proband exhibited anosmia, suggesting a possible link between *SEMA3A* gene defects and olfactory dysfunction. Extensive research has shown that SEMA3A expression increases during neuronal development in the embryonic stage (Raper 2000). The interaction between *SEMA3A* and neuropilin promotes intracellular signal transduction pathways. *SEMA3A* mRNA consists of 17 exons, encodes a protein of 771 amino acids, and contains one immunoglobulin (Ig) domain, one PSI domain, and one sema domain (Pascual et al. 2005). Here, we describe a novel variant of *SEMA3A* characterized by the deletion of exons 6–9. These exons encode the sema domain, which is critical for the interaction between *SEMA3A* and its receptors, leading to reduced *SEMA3A* levels. This discovery may provide insights into the olfactory loss symptoms observed in this pediatric patient.

We investigated the impact of the deletion of exons 6–9 in *SEMA3A* on KS, and the results suggested that there may be sex‐dependent familial variation. Further investigation into the pubertal development of family members revealed that the father initiated pubertal changes at 16 years, and the sister reached menarche at 11 years. No family member reported a history of constitutional delay in puberty. On this basis, we hypothesized that the paternally inherited gene is silenced in females, suggesting that there might be a sex‐specific expression pattern. Although the patient's sister carries the same variant, her olfactory function and pubertal development remain normal. With respect to disease incidence, the incidence of KS is higher in males than in females, with global incidence rates estimated at approximately 1:29,000 for males and 1:130,000 for females (Dai et al. 2020). Compared with females, males are more likely to exhibit disease characteristics associated with KS, even when they have an identical genetic background. In the management of KS, combination therapy utilizing human chorionic gonadotropin (hCG) and human menopausal gonadotropin (hMG) has been demonstrated to enhance the development of secondary sexual characteristics and effectively restore fertility in male patients (Gong et al. 2015; Mao et al. 2017). In this study, the patient's response to exogenous GnRH demonstrated that the sensitivity of pituitary gonadotrophs to GnRH remained largely unaffected. After three days of hCG treatment, serum concentrations of testosterone (T) and dihydrotestosterone (DHT) significantly increased, indicating that testicular function was not substantially impaired. Previous research has shown that approximately 10% of individuals with KS experience spontaneous reversal of the condition, characterized by normalized serum sex hormone levels, increased testicular volume, and the restoration of spermatogenesis following medication withdrawal. Key genes implicated in this phenomenon include *FGFR1*, *GNRHR*, *HS6ST1*, and *PROKR2* (Sidhoum et al. 2014). However, whether the symptoms of GnRH deficiency in this child are reversible requires further follow‐up and observation in the future.

The postmitotic migration of neurons to their precise destinations is crucial for the functional development of the nervous system. The development of neuroendocrine GnRH cells represents a well‐characterized instance of axophilic migration in vertebrates. GnRH neurons play an indispensable role in reproductive processes and constitute a key component of the HPG axis. In individuals with KS, abnormalities in the olfactory system, combined with the impaired migration of GnRH neurons to the brain, lead to anosmia and delayed or absent pubertal maturation.

Functional enrichment analysis in GN11 cells revealed that the genes that were differentially expressed following *SEMA3A* gene mutation were significantly enriched in pathways related to cell migration and male gonad development, as well as those associated with motor proteins and GABAergic synapses. GnRH neurons originate in the olfactory placode and migrate along the olfactory/vomeronasal nerves into the forebrain, ultimately targeting the hypothalamus, which controls gonad development (Wierman et al. 2011). Motor proteins (dynein, kinesin, myosin) are universal executors of intracellular transport and force generation, which are crucial for the migration of all neurons, including GnRH neurons (Cason and Holzbaur 2022). Binding of SEMA3A to NRP1/Plexin receptors activates downstream Rho GTPases. Rho GTPases also regulate microtubule stability and dynamics (Tivodar et al. 2015). Microtubules provide structural support and tracks for motor protein‐driven cargo transport, which is essential for sustained migration. GABA is a major neurotransmitter in the olfactory bulb and along the migratory path (Chen, Chen, Huo, et al. 2021). GABAA receptor activation can lead to Ca2+ influx. Ca2+ is a potent regulator of cytoskeletal dynamics (e.g., via calpain and CaMKII and by modulating Rho GTPases) (Khatri et al. 2019). This regulation could modulate the sensitivity or response to guidance cues such as SEMA3A. Multiple studies have shown that inhibiting GABAA receptors promotes the migration of GnRH neurons, whereas activating GABAA receptors suppresses this process (Casoni et al. 2012; Lee et al. 2008). This study further revealed that SEMA3A‐MUT neuronal cells exhibit weak migratory ability. GABAergic synaptic signaling was significantly decreased. These findings indicate that GABA serves as a critical signaling pathway through which SEMA3A modulates GN11 cell migration. In summary, we hypothesized that the SEMA3A signaling pathway may be involved in the essential migration process of GnRH neurons within the neural circuitry through cytoskeletal effectors (actin/myosin), kinesin‐related complexes (kinesin/actin/myosin), and potential regulatory mechanisms such as GABAergic signaling. Nevertheless, further investigations are needed to validate these associations. Notably, this study has certain technical limitations. While qPCR and gene panels confirmed a heterozygous deletion encompassing *SEMA3A* exons 6–9, these methods lack the resolution to map exact breakpoints or distinguish a simple contiguous deletion from a complex rearrangement (e.g., inversion‐deletion, balanced translocation). Techniques such as CNV‐seq (genome‐wide copy number detection via low‐coverage whole‐genome sequencing) or long‐read sequencing are needed to clarify the genomic architecture of this variant. Such high‐resolution mapping is clinically relevant, as complex rearrangements may involve additional genes or disrupt regulatory elements, potentially altering the phenotypic impact. In the absence of these data, we conservatively interpret this finding as a multiexon deletion while acknowledging this technical limitation.

## 5 Conclusion

In summary, this study identified a novel variant associated with KS‐specifically, the deletion of exons 6–9 in the *SEMA3A* gene within a family. This genetic defect was observed to cosegregate with the disease and is implicated in regulating cell migration and male gonad development in GnRH neuronal cell lines. Furthermore, KEGG functional enrichment analysis revealed that the underlying mechanisms involve motor proteins and GABAergic synapses. Therefore, we propose that defects in the *SEMA3A* gene may disrupt the projection and migration of the olfactory nerve, thereby affecting the proper projection and migration of GnRH neurons.

## Author Contributions

**Shaolian Zang:** formal analysis, methodology, writing – original draft. **Shasha Zhou:** data curation, formal analysis, methodology. **Qingxu Liu:** formal analysis, methodology. **Xiaoqin Yin:** supervision, writing – review and editing. **Pin Li:** formal analysis, resources, supervision, writing – review and editing.

## Funding

This work was supported by Science and Technology Commission of Shanghai Municipality (21Y21901000 and 24Y12800700).

## Ethics Statement

The studies involving humans were approved by the Medical Ethics Committee of Shanghai Children's Hospital (Approval No. 2021R092‐E03). The studies were conducted in accordance with local legislation and institutional requirements. The participants provided their written informed consent to participate in this study.

## Conflicts of Interest

The authors declare no conflicts of interest.

## Supporting information


**Appendix S1:** mgg370190‐sup‐0001‐Tables.docx.

## Data Availability

The raw sequence data reported in this paper have been deposited in the Genome Sequence Archive (Chen, Chen, Zhang, et al. 2021) in the National Genomics Data Center (Members and Partners 2022), China National Center for Bioinformation/Beijing Institute of Genomics, Chinese Academy of Sciences (GSA: CRA024728) that are publicly accessible at https://ngdc.cncb.ac.cn/gsa.
