# Supplementary material for: Identification and Functional Characterization of a Novel SEMA3A Exon Deletion Variant in Kallmann Syndrome
Source: Mol Genet Genomic Med. 2026 Jan 14;14(1):e70190. doi: 10.1002/mgg3.70190 (PMC12800911; doi:10.1002/mgg3.70190)
Supplement: Supplementary file 1 — Appendix S1: mgg370190‐sup‐0001‐Tables.docx. [file MGG3-14-e70190-s001.docx]

**Supplementary TABLE 1** Primer sequence of PCR

| Forward | Reverse |
| --- | --- |
| SEMA3A-10F GCCATTTTCACCTATGCCTT | SEMA3A-10R TCTGTCTGTAGCTGCATTGTTTT |
| SEMA3A-6F GCGAGACTTTGCTATCTTCCGA | SEMA3A-6R AACCACCTACCATTGAGCCACC |
| SEMA3A-9F CGTTTGTTCAGACTTTGCCATTCC | SEMA3A-9R CGCTTACCTGGAAGTCGTAAACAC |

**Supplementary TABLE 2** Sequences of qPCR primers

| **Gene** | **Forward primer (5’-3’)** | **Reverse primer (5’-3’)** |
| --- | --- | --- |
| SEMA3A | GGCTGGTTCACTGGGATTG | CCGTTTGCATAGTTTGCTCTGG |
| β-actin | AAGATCAAGATCATTGCTCCTCC | GACTCATCGTACTCCTGCTTGC |
| Ctnna3 | ACGCCAATAACCCTGAATATGG | TTGGAAGGATTCTGCGGACAA |
| Myh14 | CAGTGACCATGTCCGTGTCTG | CGTAGAGGAACGATTGGGCTG |
| Gabrr2 | ATGCCTTATTTGATGAGACTCGC | CCACACCTACAGGGATGGC |
| Mypn | GTGGGGACAACGAGCGAAG | GCCTCGCCAGATTGACACTT |
